# In silico biomechanical analysis of poller screw-assisted small-diameter intramedullary nail in the treatment of distal tibial fractures

**DOI:** 10.3389/fbioe.2023.1172013

**Published:** 2023-05-09

**Authors:** Jinchuan Tan, Yang Yang, Mian Wang, Xuecheng Huang, Hanbin Ouyang, Dongliang Zhao, Gang Huang, Yuping Deng, Wenhua Huang

**Affiliations:** ^1^ National Key Discipline of Human Anatomy, Guangdong Provincial Key Laboratory of Medical Biomechanics, Guangdong Engineering Research Center for the Translation of Medical 3D Printing Applications, School of Basic Medical Sciences, Southern Medical University, Guangzhou, China; ^2^ Department of Orthopedics and Traumatology, Integrated Hospital of Traditional Chinese Medicine, Southern Medical University, Guangzhou, China; ^3^ Institute of Biomedical Engineering, Shenzhen Bay Laboratory, Shenzhen, Guangdong, China; ^4^ Orthopedic Center, Affiliated Hospital of Guangdong Medical University, Guangdong Medical University, Zhanjiang, China; ^5^ Drug Discovery Center, State Key Laboratory of Chemical Oncogenomics, School of Chemical Biology and Biotechnology, Peking University Shenzhen Graduate School, Shenzhen, Guangdong, China

**Keywords:** poller screw, small-diameter intramedullary, distal tibial fractures, parallel interfragmentary motion, fracture fixation

## Abstract

**Objective:** To evaluate the biomechanical effects of Poller screws (PS) combined with small-diameter intramedullary nails in the treatment of distal tibial fractures at different locations and on different planes.

**Methods:** Nine finite element (FE) models were used to simulate the placement of the intramedullary nail (IMN) and the PS for distal tibial fractures. Structural stiffness and interfragmentary motion (IFM) through the fracture were investigated to assess the biomechanical effects of the PS. The allowable stress method was used to evaluate the safety of the construct.

**Results:** With the axial load of 500 N, the mean axial stiffness of IMN group was 973.38 ± 95.65 N/mm, which was smaller than that at positions A and B of the coronal group and sagittal group (*p* < 0.05). The shear IFM of the IMN group was 2.10 ± 0.02 mm, which were smaller than that at positions A and B of the coronal group and sagittal group (*p* < 0.05). Under physiological load, the stresses of all internal fixation devices and the nail-bone interface were within a safe range.

**Conclusion:** In the treatment of distal tibial fractures, placing the PS in the proximal fracture block can obtain better biomechanical performance. The IMN fixation system can obtain higher structural stiffness and reduce the IFM of the fracture end by adding PS.

## 1 Introduction

Tibial fractures frequently occur at the distal metaphysis, and the management of this kind of injury has been the subject of several studies. Complexity with respect to biological and biomechanical aspects, as well as surgical tools and techniques, makes the treatment of these fractures challenging and problematic (Xue et al., 2014). Due to advantages including soft tissue preservation, earlier definitive fixation, and weight-bearing, IMN has become an increasingly common method of fixation in all tibial fractures regardless of location. There are reports of high rates of malunion with IMN fixation via the traditional infrapatellar approach, 23% are associated with distal tibia fractures, poor initial reduction and IMN can not provide good stability in distal tibial fractures are reasons (Bedi and Leandkarunakar, 2006). Of the many techniques that have been described, PS is one of the methods that can be used to aid satisfactory fracture reduction and biomechanical stability (Krettek et al., 1999; Stedtfeld et al., 2004; Scolaro et al., 2016).

When using IMN to treat tibial metaphyseal fractures, the PS can reduce the width of the medullary cavity near the fracture and replace cortical bone to contact with the IMN. The PS provides the third support point for the IMN and limits the movement on the PS placement side to improve the stability. Based on several clinical studies, the PS combine with the IMN has a lower incidence of complications in bone nonunion, coronal malunion, and secondary surgery (Seyhan et al., 2012; Kulkarni et al., 2012). In an *in vitro* experiment, Baseri et al. reported that the IMN can obtain higher axial stiffness by the PS (Bedi and Leandkarunakar, 2006). However, complications related to the position of the PS were reported in clinical studies (Seyhan et al., 2012; Ricci et al., 1999). Maria et al. (2020) suggested that the improper use of the PS may not only lead to failure, but also lead to secondary damage and increase patient suffering. Therefore, whether it can be placed in a reasonable position to provide correct support for the IMN is the key to using the PS. At present, there is no biomechanical study related to the position of the PS. Therefore, it is necessary to further explore the biomechanical properties of the PS at different positions in the IMN internal fixation system.

In the current study, FE models were used to investigate whether the use of the PS technique in distal tibial fractures could provide better biological performance and structural stability. FE-based biomechanical analysis was performed given that screws were anchored in both sites of the fracture and on two anatomical planes. An allowable stress method based on our previous work (Deng et al., 2021) was used to conduct FE simulation and safety assessment. We hypothesized that the biomechanics effect of PS is related to their position and plane. Improper placement of the PS will cause nonparallel IFM of the distal tibial fracture treated with IMN. Such improper placement could potentially delay secondary bone healing by inducing nonparallel early IFM.

## 2 Materials and methods

### 2.1 FE modeling

Computed tomography was performed on a 39-year-old male cadaver. The whole tibia, distal femoral condyle, and proximal talar condyle were scanned. The slice thickness was 1.25 mm and the plane resolution was 512 × 512 pixels. By processing computed tomography slices in mimics 19.0 (Materialise, Leuven, Belgium), 3D models including the tibia, distal femur, and proximal talus were established. Point cloud data of the 3D tibia and its joint samples (talus and distal femur) were then imported into Geomagic warp 2014 (3D Systems, Morrisville, NC) to create a solid structure. A transverse 10-mm fracture space 70 mm from the distal tibial articular surface (tibia calcaneal joint) was simulated (Baseri et al., 2020).

**FIGURE 1:**
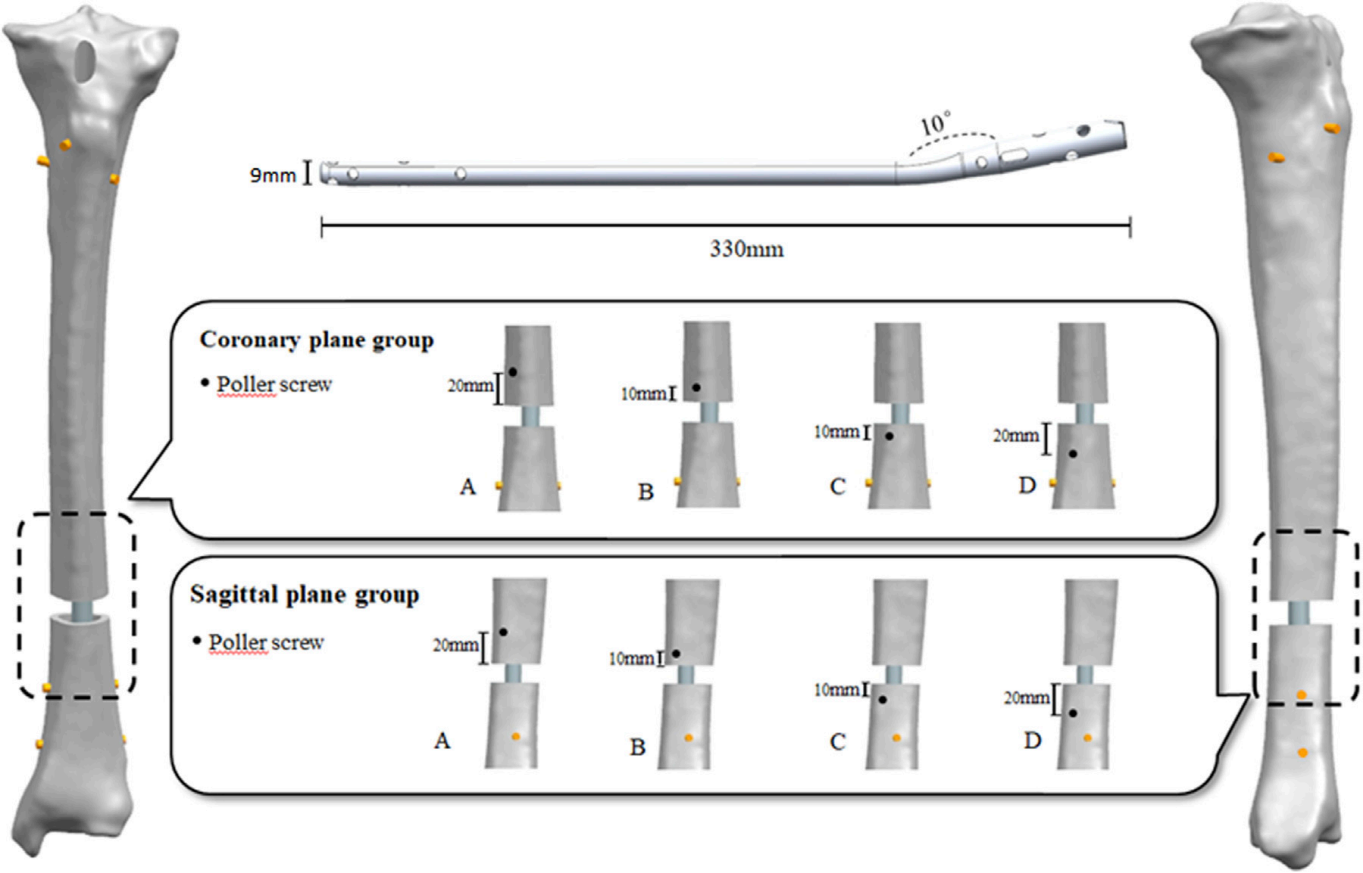
Experimental grouping and finite element model establishment.

The three-dimensional models of IMN, screws and PS were prepared in advance and imported into NX8.5 (Siemens PLM Software, Plano, Texas, United States) for assembly together with the tibial model. According to the recommendations of senior qualified surgeons, IMN with a diameter of 10 mm was selected. The distal end of the IMN was stopped at a plane, which 10 mm above the tibiotalar joint surface by simulating the clinical with frapatellar approach and placing anterogradely. The medullary cavity of tibia was expanded to make a 1 mm gap between the IMN and the cortical bone of medullary cavity. The proximal and distal of the IMN were fixed with two screws with a diameter of 4.0 mm, respectively. All screws were placed inside out, and a friction coefficient of 0.2 was used for the IMN-tibia and IMN-screw interactions (Shirazi-Adl et al., 1993). The diameter of the PS was 4.5 mm in this study and the FE models were divided into coronal group, sagittal group and IMN group. Further, we divided the coronal group and sagittal group into position A, B, C, and D according to the positions placed by the PS. The placement of PS at different positions was simulated by placing PS on both sides of the fracture line at a distance of 10 mm. The IMN group was an FE model that only used IMN for internal fixation. Considering the computational cost, all screws were simplified as cylinders for calculation. A total of nine FE models were established in this study (Figure 1).

### 2.2 Loading and boundary conditions

To apply loads and boundary conditions, the interaction surfaces of proximal talus, distal tibia and distal femur proximal tibia were considered. These surfaces were then used to set boundary conditions such as where the load is applied to the proximal tibial meniscus and the range of motion of all nodes on the distal tibial ankle surface is limited. The FE model was loaded under axial force of 500 N, to simulate the physiological load of human standing, and the axial force was distributed in 60% of the medial plateau and 40% of the lateral tibial plateau (Raja Izaham et al., 2012a). IMNs, PSs, and locking screws were considered titanium alloys and modeled as equal diameter, linear elastic, and homogeneous materials with Young’s modulus of 110,000 MPa and a Poisson’s ratio of 0.3. Each part of the tibia was considered to be isotropic homogeneous material and the Young’s modulus was assigned (Table 1). Screws were tied to adjacent bones in all degrees of freedom. For simplicity, a contact case with a penalty function equal to 0.2 is defined at the interface between nail and bone, PS and nail. Notably, there may be initial gaps at the IMN bone and PS nail interfaces, which may contact each other during loading and simulation. All samples were meshed with 10-node tetrahedral elements (Table 2).

**TABLE 1:** Material properties used in FE analysis and interaction.

Materials	Young’s modulus (MPa)	Poisions’s ratio
Cortical bone	8,000	0.3
Cancellous bone	1,500	0.2
Titanium alloy	110,000	0.3

**TABLE 2:** Number and type of elements used in the FE models of this study.

Parts	Element type	Number of elements	Number of nodes	Approximate element size (mm)
Tibia-I	C3D10	150,693	238,965	0.8–4.0
Tibia-P	C3D10	162,563	257,095	0.8–4.0
IMN	C3D10	65,195	108,819	0.6–3.0
Screws	C3D10	5,777	9,296	0.8
Poller screw	C3D10	6,783	10,805	0.6

Tibia-I: tibial models of the IMN, group; Tibia-P: tibial models of the coronal group and sagittal group.

### 2.3 Mechanical experiment validation

**FIGURE 2:**
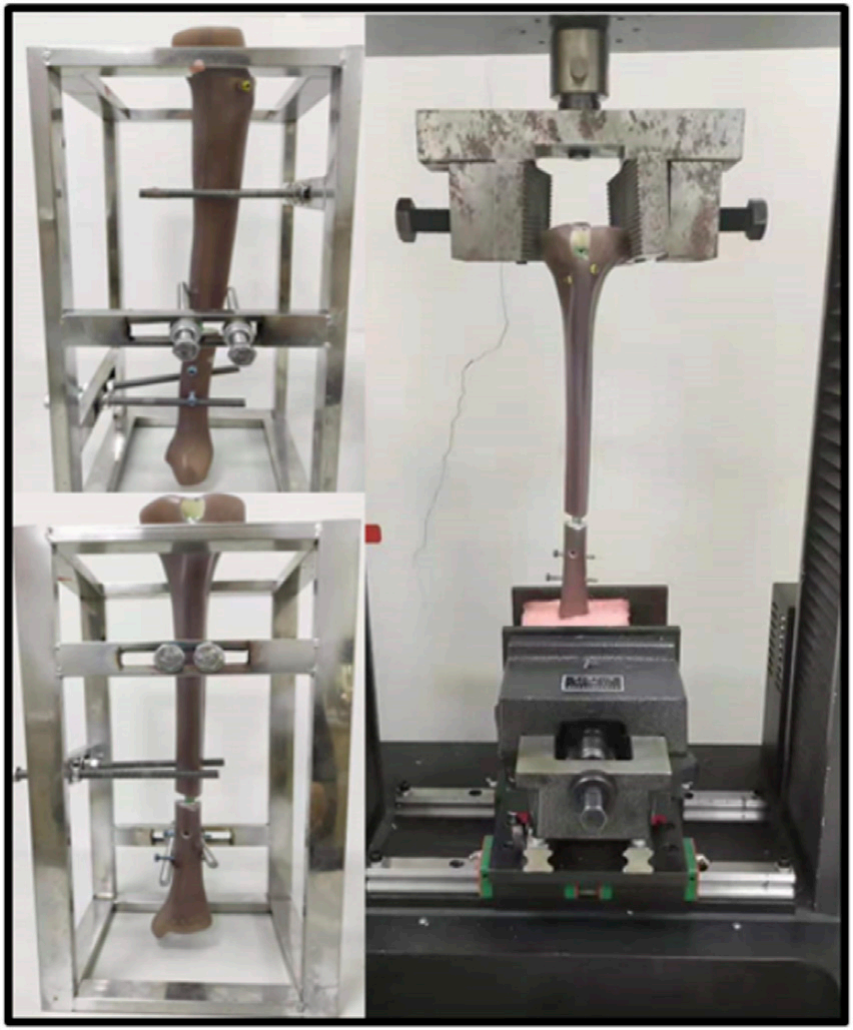
Fourth generation composite tibias and electronic universal testing machine (A TES6010, China).

This study was verified by literature and biomechanical experiments. The same FE modeling method was used to carry out biomechanical experiments on three-fourth generation composite tibias (Sawbones, Pacific Research Laboratories Inc., Vashon Island, WA, United States) (Figure 2). The effectiveness of the FE method is verified by comparing the axial stiffness of FE model and composite tibia. The specific mechanical experiment verification method is as follows.

The axial stiffness tests were conducted by an electronic universal testing machine (A TES6010, China). The specimens were first pre-loaded with the testing machine in position to control a vertical load of up to 100 N. And then the loader was controlled to press down at a rate of 1 mm/min until the force is up to 1000 N. Loads and displacements were recorded and for at least 90 s after load removal for each repetition. Three loading repetitions were performed for each loading configuration, with the specimen being allowed to recover at least 10 min between replicates. Loads and displacements were collected by sensors. According to the load-displacement data, the slope of the linear region was calculated as the axial stiffness of the sample.

### 2.4 Data analysis

**FIGURE 3:**
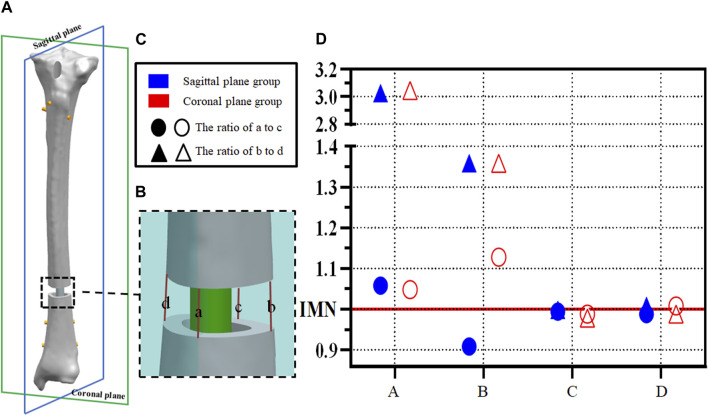
**(A,B) **Example diagram of fracture space symmetry analysis; **(C,D)** Point distribution of fracture space symmetry analysis.

In order to evaluate the IFM, the fracture gap was marked on the coronal plane and sagittal plane, and the length of the mark was measured to reflect the IFM of the fracture space (Figures 3A, B). Nodes of the marks on the FE model are pre-labeled for the calculation of line lengths. To better evaluate the effects of the PS on secondary bone healing, the parts of the sagittal plane passing through the fracture space were marked “a” for anterior and “c” for posterior at the outer edges of the tibial cortical bone. Similarly, the part of the coronal plane passing through the fracture space was divided into “b” for lateral and “d” for medial. Statistical analysis of the ratio of a and c and the ratio of b and d was used to calculate the symmetry of the fracture space, to better reflect IFM.

Nine static FE simulations were performed in the current study using the Abaqus/Standard 2017 (Dassault Systèmes, France) software. The equivalent stress and the positions of the model and template were used in the output measurement of statistical analysis. Mean stiffness and displacement values derived from the two models were compared via one-way analysis of variance, and significant results were followed by a *post hoc* test with Bonferroni correction. *p* < 0.05 was considered statistically significant.

## 3 Results

### 3.1 Structural stiffness and shear displacement of the fracture site

**FIGURE 4:**
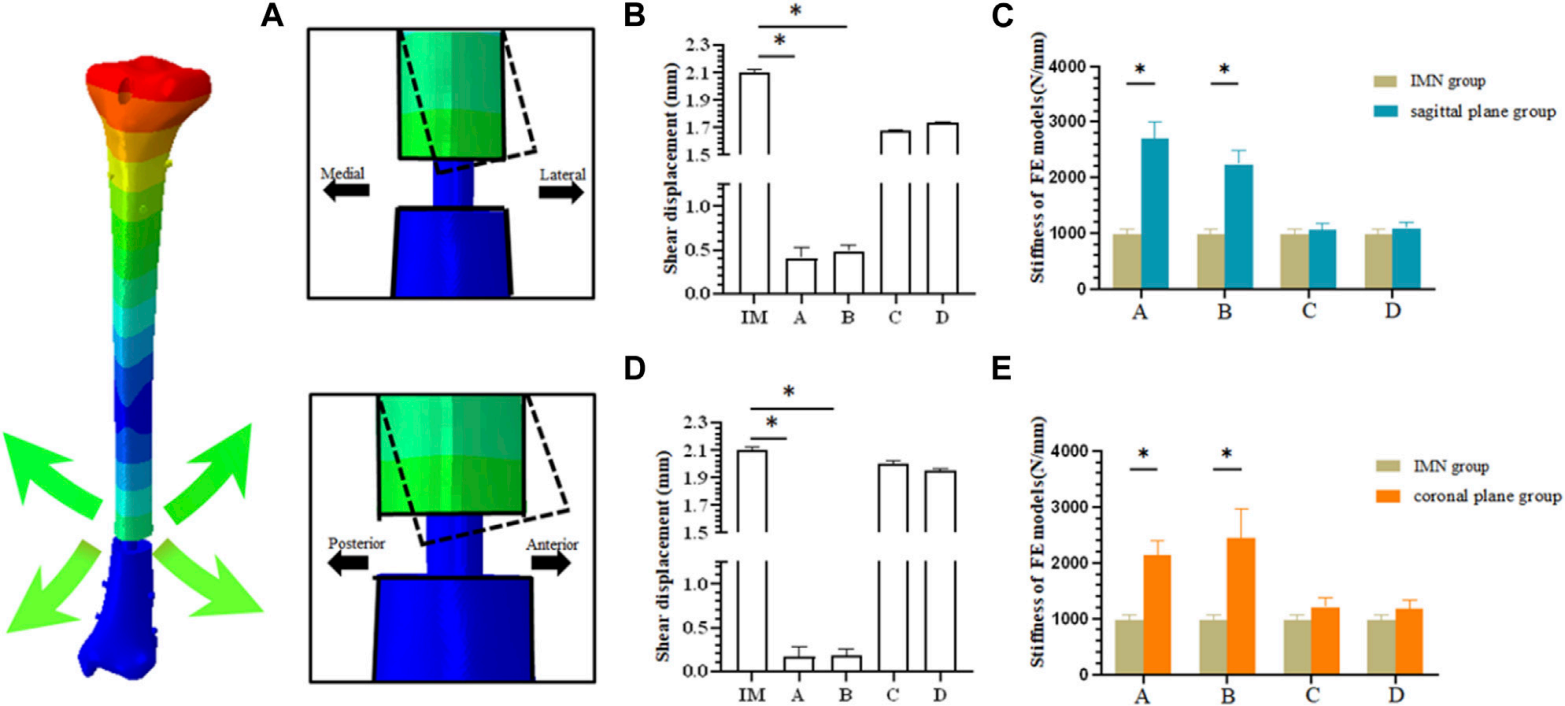
**(A)** Schematic diagram of fracture end displacement; **(B)** Shear displacement between fracture blocks in sagittal plane group; **(C)** Comparison of axial stiffness between sagittal plane group and IMN group; **(D)** Shear displacement between fracture blocks in coronal plane group; **(E)** Comparison of axial stiffness between coronal plane group and IMN group.

The stiffness derived from the FE model was consistent with the experimental stiffness in normal models (Lee et al., 2014a; Chen et al., 2018; Baseri et al., 2020). Under the load condition of 500N, the mean axial stiffness with IMN only was 973.38 ± 95.65 N/mm, which was significantly less than that in the two PS groups at positions A and B. The axial stiffness means in the sagittal plane group from position A and B were 2,698.84 ± 296.53 N/mm and 2,234.47 ± 254.69 N/mm (*p* < 0.05) (Figure 4C). The axial stiffness means in the coronal plane group from position A and position B were 2,139.50 ± 262.46 N/mm and 2,439.53 ± 518.26 N/mm (*p* < 0.05) (Figure 4E). There was no difference between coronal and sagittal groups. In the sagittal plane group, the shear displacement of position A was 0.410 ± 0.12 mm. The value of the shear displacement of position B was 0.487 ± 0.06 mm. The shear displacements of positions A and B in the coronal plane group were 0.168 ± 0.11 mm and 0.187 ± 0.07 mm, respectively. The above displacements were all smaller than the shear displacements of the models without PS: 2.10 ± 0.02 mm (*p* < 0.05) (Figures 4A, B, D).

### 3.2 Parallel IFM

Axial displacements located at a,b,c, and d were calculated between cortices at the fracture site (Figures 3A, B). The ratio of a to c and the ratio of b to d was calculated to indicate the symmetry of the axial displacement at the plane fracture site. An IFM ratio range from 0.90 to 1.10 is usually considered to represent parallel IFM (1.00 represents completely parallel IFM at the fracture site (Bottlang et al., 2010; Lujan et al., 2010; Bottlang et al., 2015; Bottlang et al., 2016). In the IMN model, these two ratios are 0.99 and 1.00, respectively. However, the ratios of model A and model B are more dispersed in both the sagittal plane group and coronal plane group. The ratio of B to D showed significant changes, 3.03 and 1.36 in the sagittal plane group and 3.05 and 1.36 in the coronal plane group, respectively (Figure 3D).

### 3.3 Mechanical experiment validation

**FIGURE 5:**
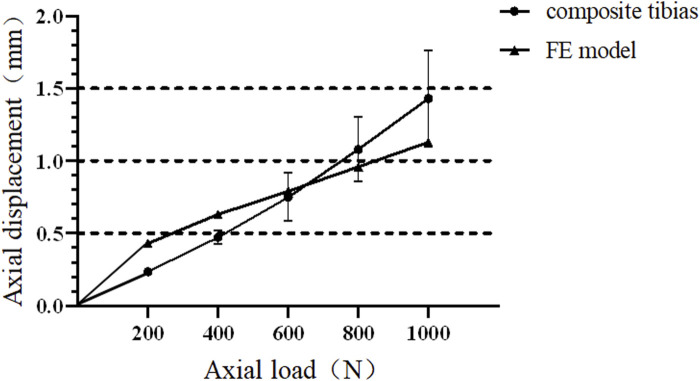
Force and displacement curves of composite tibia and finite element model under axial compression.

The biomechanical experimental validation results are presented in Figure 5. In the process of axial compression load from 0 N to 1000 N, the axial stiffness of the mechanical experiment and FE model group are 838.36 ± 150.52 N/μm and 996.26 ± 139.87 N/μm, respectively.

### 3.4 Safety assessment

In all nine FE models, the maximum stress was evident at the junction of the locking nail and the IMN. The maximum stress of the PS was located at position B in the sagittal plane group under a load of 500 N, and it was 57.78 MPa. The minimum stress was 31.78 MPa, and it was located at position D in the coronal plane group under a load of 500 N.

When evaluating the structural stability associated with using the PS technique in distal tibial fractures with IMN fixation, we also evaluated the risk of a fracturing of the screws that exhibited the maximum deformation in each model. According to our previous work, the allowable stress of the medical titanium alloy can be calculated using the following formula:

$$\left[\sigma \right]=\frac{\sigma_{Y}}{n}$$

Thus, the maximum safe stress of the IMN and screws was calculated as:

$$\left[\sigma \right]=412.5\;MPa$$

**FIGURE 6:**
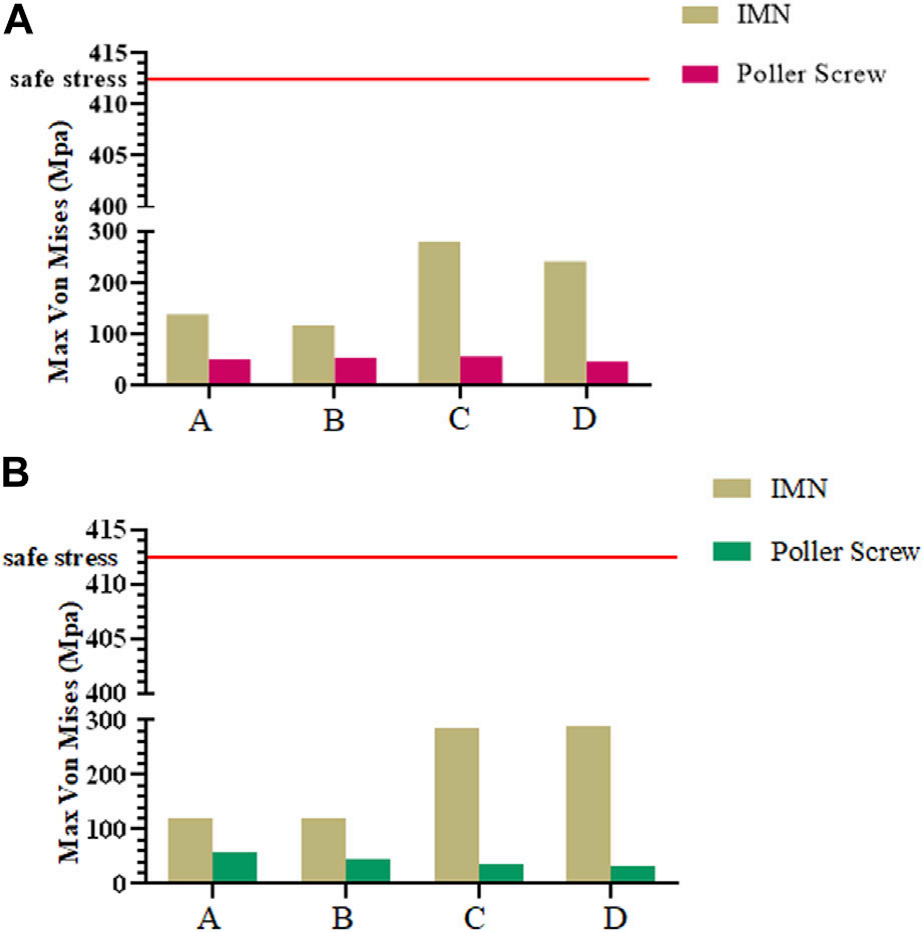
**(A)** Maximum Mises stress of IMN and PS in sagittal plane group; **(B)** Maximum Mises stress of IMN and PS in coronal plane group.

The maximum von Mises stress should be less than the allowable stress to ensure structural safety. The maximum von Mises stresses of the models under axial compression loading compared with the allowable stress are shown in Figures 6A, B.

## 4 Discussion

The results of this study support the hypothesis that the biomechanical characteristics of PS were related to the position and the plane. We analyzed the axial stiffness obtained by placing PS in different planes and positions, and evaluated the IFM and safety of PS combined with IMN internal fixation systems for distal tibia fractures.

PS was proposed by Krettek et al. (1999b), and then applied to the clinical treatment of the tibial metaphyseal fracture. Then in 2004, Stedtfeld et al. (2004) simulated the placement of more kinds of PS by establishing a simple model, and further elaborated the mechanical effect of the PS. Hannah et al. (2014) proposed a more detailed clinical application method for PS in 2014. However, the above studies did not further elaborate on the biomechanical properties of the PS. In the current study, FEA was used to study the influence of specific factors in a given system to better understand the role of PS in internal fixed structures (Baseri et al., 2020). FEA can effectively focus on a single factor and offset the influence of other variables, while clinical research may be affected by several controlled and uncontrolled variables. Therefore, based on the 3D model of the proximal tibial extraarticular fracture, using FEA to evaluate the placement of PS in different positions and planes is helpful to further clarify the biomechanical mechanism.

In clinical reports, PS users were concerned about whether the PS can provide additional stability during treatment and achieve satisfactory reduction results. A number of clinical studies have shown that the use of the PS can reduce the probability of complications such as nonunion (Maria et al., 2020). Our study showed that the axial stiffness of the coronal group and sagittal groups was improved compared with the IMN group, and the stiffness of position A and B was significantly increased. It suggested that the PS could bring additional stability to IMN, which is the same as the results of (Baseri et al., 2020). We also found that the stiffness obtained by placing the PS at the distal end of the fracture was lower than that by placing PS at the proximal end of the fracture. This result indicated that the position of the PS could affect the stability of the internal fixation, which has been partly demonstrated by the study of (Seyhan et al., 2012). In this study, the FE model simulated the comminuted fracture of the distal tibia, and the force line has been completely corrected, so it was diffcult to evaluate the role of the PS in the fracture reduction process. Compared with the clinical application, the placement of internal fixation in the model is more ideal. In addition, the setting of boundary conditions makes the displacement of the distal tibial fracture block decrease in the whole loading process. Based on the above reasons, the distal fracture block will not be greatly affected during model loading. Under physiological conditions, the force on the proximal tibia is nonparallel, and the internal and external forces are distributed in 60% and 40% (Chen et al., 2018). When the IMN could not form good contact with the isthmus of the tibia, such as the space that may be generated after reaming or the use of a small-diameter intramedullary nail for the treatment of fractures, this asymmetry will be more obvious, which may be one of the reasons why the PS placed in the proximal fracture block can obtain better axial stiffness. Although the differences in the anatomical morphology of the medullary cavity and the gap between the IMN and the medullary cavity may lead to changes in the axial stiffness of internal fixation, it can still show that PS plays a constructive role in maintaining the alignment of nails in the medullary cavity, thereby enhancing the stability of the bone-implant structure (Figures 4C, E). The results of this study, corresponding to the axial stiffness of the bone-implant construct, are in agreement with the results of Hoegel et al. (2012), Baseri et al. (2020).

The decrease in IFM is another change observed. As a mechanical stimulation that directly affects bone healing, IFM is one of the main causes of poor fracture alignment and affects the healing of distal tibial fractures (Claes et al., 1998; Perren, 2002; Raja Izaham et al., 2012a; Samsami et al., 2019). Lindvall et al. (2009) reported that the incidence of nonunion caused by improper reduction of IMN was higher than that caused by locking plate, and considered that may be due to shear movement at the end of fracture space. In this experiment, when PS was added, the IFM of all FE models decreased, which is consistent with the results of Amin (Baseri et al., 2020). The addition of PS can effectively limit the shear displacement of the fracture end and provide more stable conditions for the healing of the fracture end.

However, The effect of the PS on the parallel IFM in distal tibial fractures should also be concerned. Parallel IFM can further affect the formation of callus. Bottlang et al. (2015) found through animal experimental research that symmetrical axial movement can increase the healing strength by 54% and load-bearing capacity by 156%. For the intramedullary fixation mode highly dependent on secondary bone healing, the parallel IFM is one of the factors that should be considered. Our study observes the symmetry of IFM by extracting the length of the preset line segment and calculating the proportion of the corresponding line segment. In the IMN group, the symmetry of IFM is consistent, and the ratio between two pairs of lines was 0.99. In the coronal plane group and the sagittal plane group, the maximum value of 3.03 can be found, which appears in the ratio of b and d of the model A in the coronal plane group. The minimum value is 0.92, which appears in the ratio of a to c of the sagittal group B model. It is worth noting that by observing the ratio of model b to d, the asymmetry of IFM is mainly concentrated on the coronal plane (Figure 3D). The above results show that the addition of PS can trigger the asymmetry of IFM and this phenomenon is more easily observed in the coronal plane. Radiological observation of PS involved in the fixation of patients with callus growth is nonparallel and coronal deformity is more often reported (Maria et al., 2020). In addition, in all FE models, the maximum stress generated by the PS is 57.88 MPa, which is a safe value. However, when the PS is placed on the proximal fracture block, the stress of the whole internal fixation system shows a concentrated trend on the contact surface between IMN and PS, which also poses a certain challenge to the long-term safety of this internal fixation system. Clinically, stress concentration and damage to cell resources in bone marrow and periosteum during PS insertion should be considered adverse effects on its application. In the bone nail structure. It is well known that the lack of cellular resources at the fracture site can lead to delayed healing or even nonunion (Norio Sonoda, 2003). Therefore, when PS is used, can be considered as another reason for the delay or non healing of the healing process.

The current study had some inherent limitations. First, the FEA was only based on the CT data of one 39-year-old male cadaver, even though this cadaver was carefully selected and can be regarded as representative in terms of age and bone quality. Second, the force and contributions of the ligaments and muscles were ignored, which is not the most advanced loading analysis. Third, the material properties assigned to cortical bone and cancellous bone may not reflect those of actual bone. Finally, in this study, we only analyzed the vertical load of the tibial platform but not the torsional load. In the future, a more comprehensive analysis will be conducted.

## 5 Conclusion

This study confirmed that in the treatment of distal tibial comminuted fractures that do not involve the ankle surface, the insertion of PS at the proximal of fracture site can obtain higher structural stiffness and effectively reduce the shear IFM. However, clinical users should also consider the effect of changes in motion of non parallel IFM on secondary fracture healing and the potential risk of stress concentration after PS.

## Data Availability

The original contributions presented in the study are included in the article/Supplementary Material, further inquiries can be directed to the corresponding authors.

## Abbreviations

FE, finite element; FEA, finite element analysis; IFM, interfragmentary motion; IMN, intramedullary nail; PS, Poller screws.
